# Simulation-based education to improve management of refractory anaphylaxis in an allergy clinic

**DOI:** 10.1186/s13223-023-00764-9

**Published:** 2023-01-29

**Authors:** Ana M. Copaescu, Francois Graham, Nathalie Nadon, Rémi Gagnon, Arnaud Robitaille, Mohamed Badawy, David Claveau, Anne Des Roches, Jean Paradis, Matthieu Vincent, Philippe Bégin

**Affiliations:** 1 Department of Medicine, Allergy-Immunology Division, Université de Montréal, Montreal, QC Canada; 2 Department of Medicine, Division of Allergy and Clinical Immunology, McGill University Health Centre (MUHC), McGill University, Montreal, QC Canada; 3 Department of Medicine, Université de Montréal, Montreal, QC Canada; 4 Learning and Simulation Center, CHUM Academy, Montreal, QC Canada; 5 Department of Medicine, Allergy-Immunology Division, Université Laval, Quebec, QC Canada; 6 Department of Anesthesiology, Université de Montréal, Montreal, QC Canada; 7 Department of Anesthesiology, Montreal Neurological Institute and Hospital, Montreal, QC Canada; 8 Department of Emergency, Université de Montréal, Montreal, QC Canada; 9 Department of Emergency, Hôpital Charles-Le Moyne, Université de Sherbrooke, Greenfield Park, QC Canada; 10 Department of Emergency, CHU Sainte-Justine, Université de Montréal, Montreal, QC Canada

**Keywords:** Medical education, High fidelity simulation based-learning, Anaphylaxis, Allergic emergencies, Anaphylaxis management, Teamwork, Allergy clinic, Ambulatory setting

## Abstract

**Background:**

High-fidelity simulations based on real-life clinical scenarios have frequently been used to improve patient care, knowledge and teamwork in the acute care setting. Still, they are seldom included in the allergy-immunology curriculum or continuous medical education. Our main goal was to assess if critical care simulations in allergy improved performance in the clinical setting.

**Methods:**

Advanced anaphylaxis scenarios were designed by a panel of emergency, intensive care unit, anesthesiology and allergy-immunology specialists and then adapted for the adult allergy clinic setting. This simulation activity included a first part in the high-fidelity simulation-training laboratory and a second at the adult allergy clinic involving actors and a high-fidelity mannequin. Participants filled out a questionnaire, and qualitative interviews were performed with staff after they had managed cases of refractory anaphylaxis.

**Results:**

Four nurses, seven allergy-immunology fellows and six allergy/immunologists underwent the simulation. Questionnaires showed a perceived improvement in aspects of crisis and anaphylaxis management. The *in-situ* simulation revealed gaps in the process, which were subsequently resolved. Qualitative interviews with participants revealed a more rapid and orderly response and improved confidence in their abilities and that of their colleagues to manage anaphylaxis.

**Conclusion:**

High-fidelity simulations can improve the management of anaphylaxis in the allergy clinic and team confidence. This activity was instrumental in reducing staff reluctance to perform high-risk challenges in the ambulatory setting, thus lifting a critical barrier for implementing oral immunotherapy at our adult center.

## Background

High-fidelity simulations based on real-life clinical scenarios have an essential role in medical education. They are frequently used to improve patient care, knowledge and teamwork in different acute care settings such as the intensive care unit or the emergency room [1–5]. Simulations provide a safe environment where clinicians can practice specific skills for acute events and build their confidence while standardizing management [1]. This is particularly useful when preparing for rare events seldom encountered in the clinic that can lead to critical consequences if not appropriately managed.

During their training, Canadian allergy-immunology physicians are well prepared to manage anaphylaxis that responds to epinephrine [6]. However, even well-trained and experienced clinicians have little hands-on experience in the management of severe refractory cases of anaphylaxis. As with any office medical emergency, these can represent a significant source of anxiety for medical and administrative personnel [4]. Simulation-based medical education allows trainers to develop specific skills without exposing patients to avoidable errors [2, 7]. In addition to clinical knowledge and skills, medical simulations have also been shown to improve non-cognitive abilities such as teamwork, leadership and communication skills [5, 8–10]. This is highly relevant given that studies have shown that the leading causes for poor patient care in a general practitioner’s office are (1) lack of communication between staff members, and (2) lack of equipment and organization in the clinic [1]. Post-simulation surveys have shown that such activities are generally well accepted and appreciated by participants [11, 12].

A high-fidelity simulation-based training for advanced anaphylaxis life-support was developed and implemented at our center, including high-fidelity mannequins and simulated patients in an adult allergy outpatient clinic. This initiative was motivated by a change in the clinic environment with the arrival of new staff and changes in clinical practice that some staff members were less comfortable with, such as oral immunotherapy and direct penicillin challenges without skin testing. Our main goal was to assess if critical care simulations in allergy improved performance in the clinical setting. Furthermore, we sought to determine the staff members’ perceptions of the improvement in team performance and the safety of anaphylaxis management in the allergy clinic following the simulation training.

## Methods

### Participants

In January 2018, all staff members from the allergy clinic at the Centre Hospitalier de l’Université de Montréal (CHUM) in Montreal, Canada, including physicians, allergy-immunology fellows and allergy nurses, were invited to participate in a simulation activity. The activity was repeated in July 2018 with new allergy-immunology fellows, nurses, and allergy-immunologists who had missed the first activity. Both simulations had identical clinical scenarios, were completed in the same environment and required the same equipment. The institutional ethics committee approved the research project, and all participants signed informed consent.

### Clinical scenarios

**Fig. 1:**
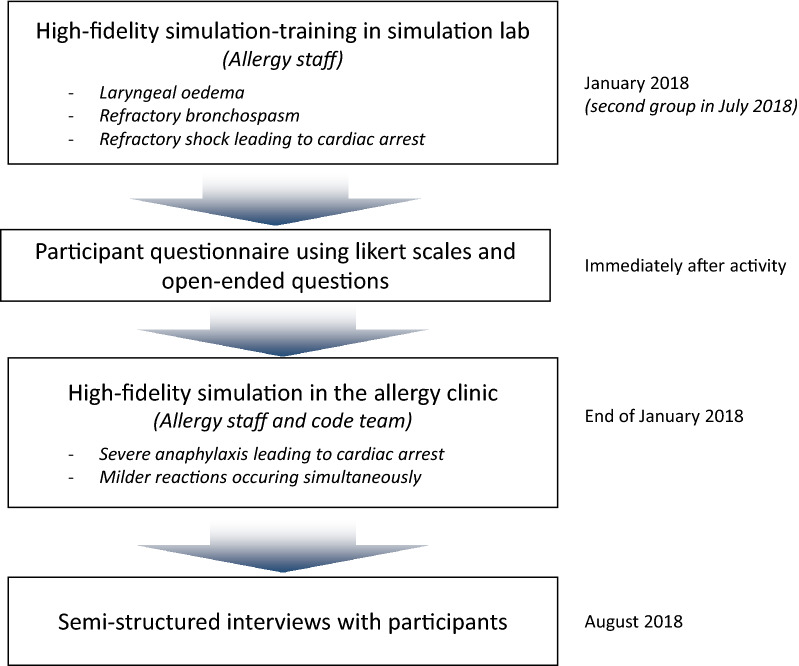
Sequence of activities

Advanced anaphylaxis life support adult scenarios were developed by a multidisciplinary panel of simulation experts, including two anesthesiologists, two emergency physicians, two intensive care specialists, and two allergy immunologists. The specific educational objectives of each scenario were based on the competency framework from the Crisis Resources Management (Royal College of Physicians and Surgeons of Canada) [13] as well as the Canadian Medical Education Directions for Specialists (CanMEDS) [14] and the School of nursing professional practice framework (*Faculté des sciences infirmières de l’Université de Montréal*). The team ensured that the custom-designed scenarios had the necessary complexity, responded to specific objectives, and covered the topics appropriately. The sequence of activities is illustrated in Fig. 1.

The first part of the program took place in the high-fidelity simulation-training laboratory at the CHUM. This state-of-the-art multidisciplinary simulation workshop focused on updating the participants’ competencies. Participants were divided into groups of 4 to 5 people. They attended three allergy scenarios of 10–15 min each designed to review specific emergencies that could occur in the clinic: (1) laryngeal angioedema, (2) refractory bronchospasm in a patient with aspirin-exacerbated respiratory disease, and (3) refractory shock leading to cardiac arrest. In the third scenario, participants were expected to repeat the epinephrine dose, administer intravenous fluid, monitor vital signs and initiate advanced cardiovascular life support while waiting for the code team. They were not expected to secure the airway or start the infusion of inotropes themselves but had to assist the code team. The actor leading the code played the role of an ICU fellow with high technical skills who had never treated anaphylaxis in the past. For example, he would ask the participants’ advice on possible alternatives to epinephrine. In the bronchospasm scenario (#2), he would ask the participants which induction agent to use for endotracheal intubation. Furthermore, unless the participants realized the ICU fellow was not providing sufficient expiratory time, the patient would evolve to obstructive shock from hyperinflation.

Before the simulation, the groups were given time to familiarize themselves with the simulated environment, the mannequin, the equipment and the available mock medication. Before each scenario, the participants received pre-briefing instructions regarding their roles (managing physician, second available physician, nurse, respiratory therapist, patient, etc.) and a written clinical script that introduced the scenario (e.g. location, information about the patient, etc.). Allergy-immunologists and allergy-immunology fellows took turns playing a physician or a support role while the nurses kept playing their roles. The participants were asked to behave as they would in a real-life situation but within their given role (e.g. the attendant playing the role of the patient would not provide advice on medical management). The simulation environment was meant to reproduce the outpatient allergy clinic in terms of seating, such as chairs and stretchers, medical equipment and available drugs. In this context, as mentioned, the participants were not expected to perform advanced airway interventions such as intubation or start inotrope infusion. Still, they were expected to administer medications to treat anaphylaxis and call for help. Depending on the specific scenario, an actor-patient, a high-fidelity simulator mannequin or both were used.

A faculty supervisor and a simulation technologist observed the participants behind a one-way mirror during each simulation. They controlled the progression of the clinical scenario, the mannequin’s voice, and its vital signs based on the actions of the participants involved in the case. A pre-determined algorithm was used to help make proper adjustments according to the learner’s performance. For example, the algorithm would indicate how to change vital signs parameters after 5 min depending on whether or not intramuscular epinephrine had been administered.

Each simulation was immediately followed by a debriefing session with the faculty (one debriefing session for each scenario). These meetings lasted 20 min, covering various aspects of performance and team dynamics as well as the scenario’s specific objectives.

The second part of the program consisted of an *in-situ* allergy clinic simulation with three actors and one high-fidelity mannequin controlled remotely by a simulation technician. The goal of this simulation was to test processes in a hospital, including the code team, the hospital security and the non-medical personnel. During the simulation, there were no visual aids, such as anaphylaxis management posters previously available in the clinic, that could have impacted the participants’ performance. To reflect the real-life environment, the team had to manage multiple co-occurring events, including milder reactions and anxiety attacks, with one of the patients progressing to acute respiratory failure and hemodynamic instability secondary to anaphylaxis. At that point, the high-fidelity mannequin replaced the actor. The team also had to initiate resuscitation while waiting for the code team. The participants knew which week but not what day the simulation would take place. One physician and two nurses were designated to play active roles, while the rest of the participants from the first part were silent observers. The faculty supervisor observed the participants in the treatment room and relayed information to the simulation technologist, who controlled the mannequin from an adjacent room, following a pre-determined algorithm. A second faculty supervisor would remain in the main challenge room to guide the actors and the nurse that managed the milder reactions. The activity was also followed by a debriefing session led by simulation experts to which all staff members were invited. Issues such as refractory anaphylaxis management, crisis management, medication storage and doses, patient transport and logistics were explicitly discussed. Gaps in the process were identified, and remediation solutions were suggested during the feedback session.

### Data collection and analysis

**Fig. 2:**
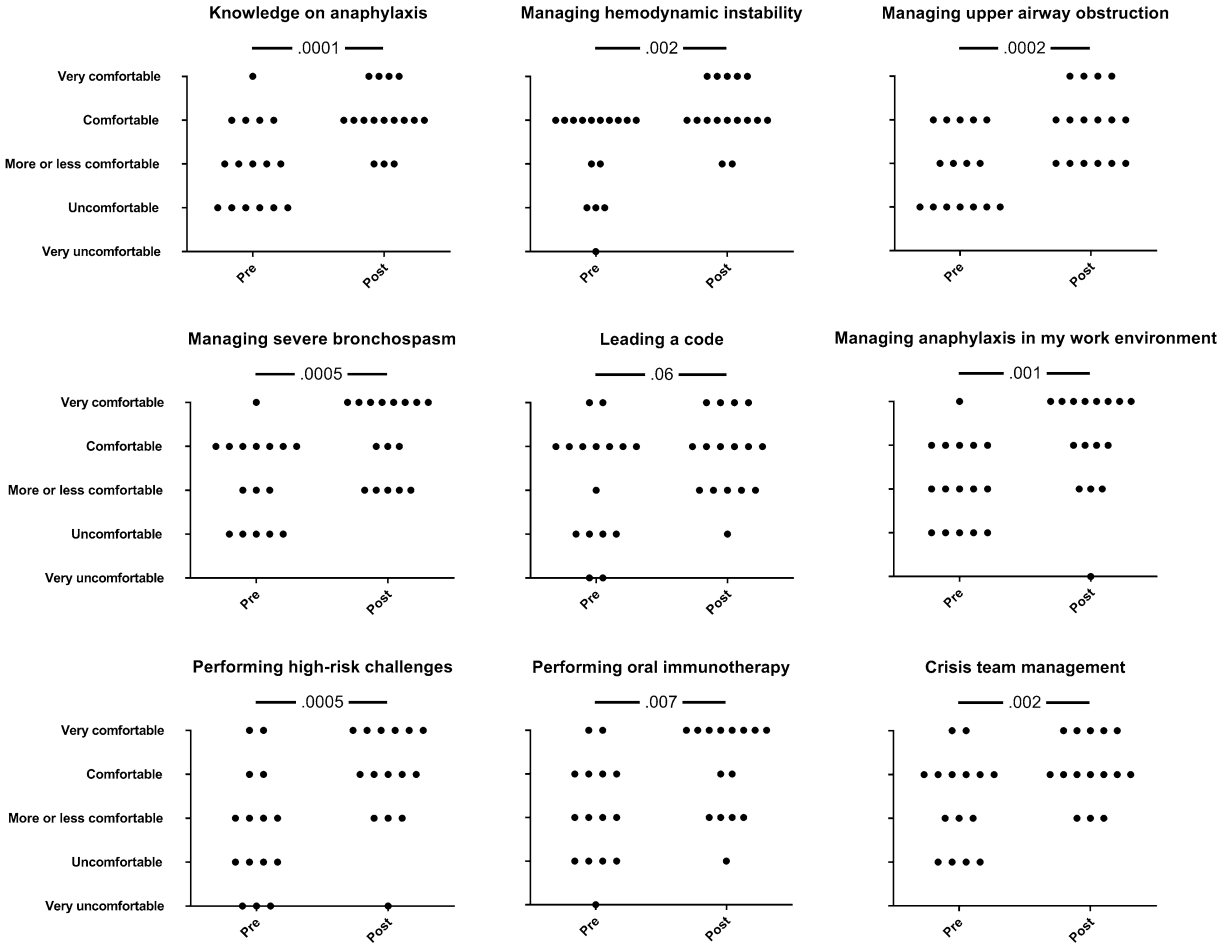
Variation in confidence level before and after the simulation activity. Dots indicate individual participants’ answers. *P*-values were calculated using the Wilcoxon matched-pairs signed rank test

**Fig. 3:**
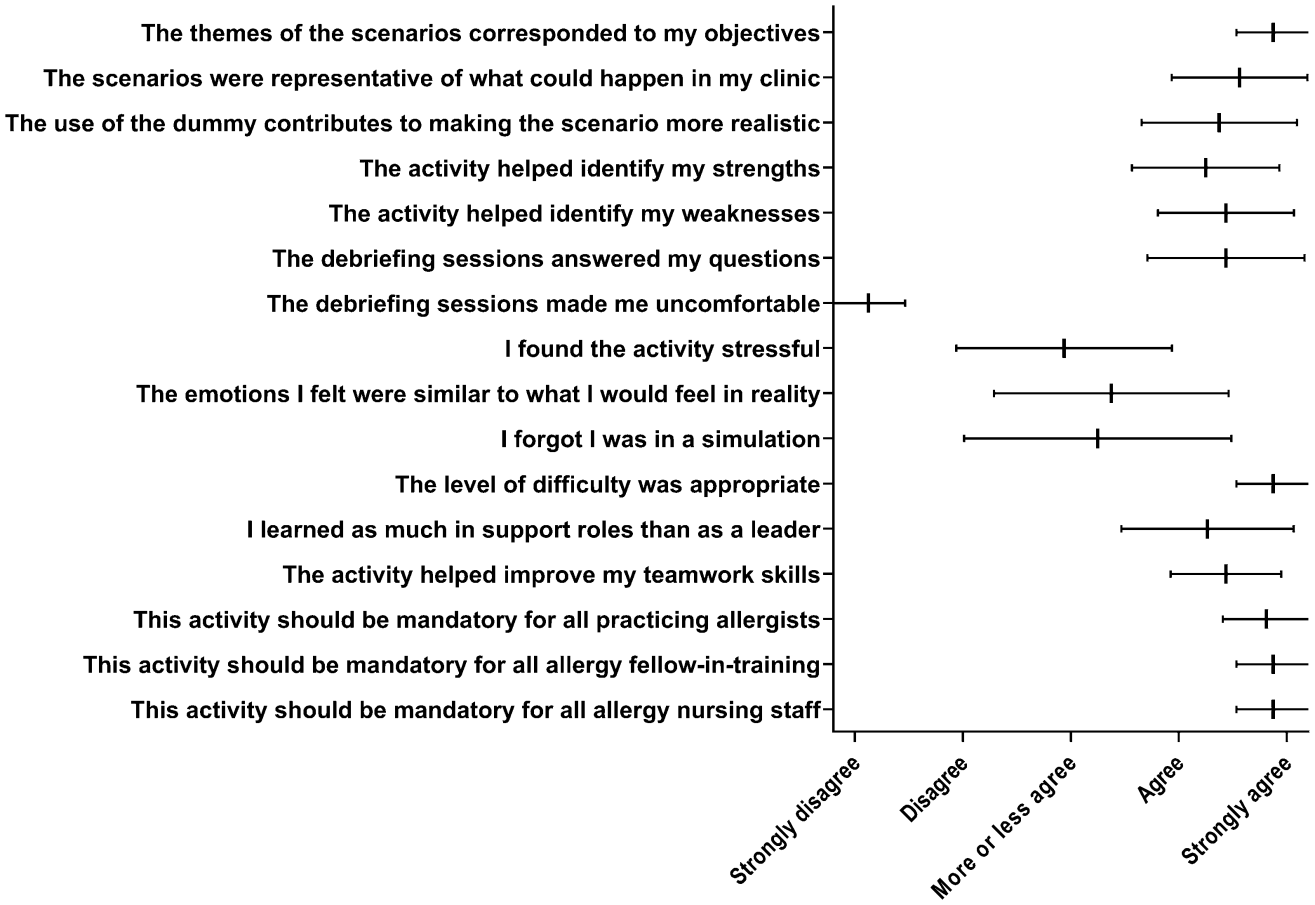
Participant’s feedback. The error bars correspond to the 95% confidence interval around the average response

After the laboratory simulation activity, the 17 participants filled out an anonymous paper questionnaire composed of four parts. The first section consisted of demographic variables. The second section included nine questions aiming to compare the participant’s confidence in their ability to manage similar real-life scenarios before and after the activity, with 5-point Likert scales (Fig. 2). This was followed by 18 sentences providing feedback on the experience using a 5-point Likert scale (Fig. 3). The last section consisted of two open-ended questions asking for strengths and means to improve the activity.

Six months following the activity, participants who had managed real cases of anaphylaxis in the clinic following the activity were invited to participate in semi-structured qualitative interviews. The same researcher conducted the interviews in French and recorded them for subsequent analysis. We used four open-ended questions, and the interviewer could reformulate them up to three times to allow participants to express their opinions. The first question asked what they thought of the simulation activity in general. The second one specifically addressed the management of real-life anaphylactic reactions. The third question asked what had been the impact of the simulation training on the management of these cases. The last question asked what they thought were the current strengths and weaknesses of anaphylaxis management in the allergy clinic.

### Outcomes

The primary outcome was to assess if critical care simulations in allergy improved the participant’s perceived performance in the clinical setting (quantitative and qualitative questionnaires). Two secondary outcomes aimed to (1) analyze staff members’ perceptions on the improvement of team performance using quantitative and qualitative questionnaires (5-point Likert scale and open questions) and (2) evaluate the impact of a simulation activity on the clinical management of anaphylaxis using semi-structured qualitative interviews.

### Analyses

Questionnaire answers were analyzed using descriptive statistics. Pre-post changes following the simulation were compared using Wilcoxon signed-ranked test (Graphpad Prism 6). Interview themes based on the existing literature were identified by two researchers based on interview transcripts. The inductive method using thematic content exploration was used to analyze the interview transcripts to identify common themes and patterns across the data set. Categories were established by consensus between the two researchers.

## Results

### Demographics

Four nurses, seven allergy-immunology fellows and six allergy/immunologists (17 participants) underwent the simulation. Among the fellows, 4 (57%) were starting their allergy-immunology training, and 3 (43%) had more than one year of training. The participants’ demographic characteristics are shown in Table 1. Most participants (59%) had little experience in simulation. Two participants reported that they were managing more than 20 anaphylactic reactions per year, while the majority (65%) managed between 1 and 10 anaphylactic reactions per year.

**Table 1:** Demographic characteristics (*N* = 17)

Variables	*N* (%)
Sex (female)	11 (65)
*Age (years)*	
≤ 45	10 (59)
> 46	7 (41)
*Practice setting*	
Community clinic	0 (0)
University Hospital (outpatient clinic)	17 (100)
*Position*	
Physicians	6 (35)
A-I fellows	7 (41)
Nurses	4 (24)
*Years of practice*	
≤ 10	9 (53)
11–20	2 (12)
≥ 20	6 (35)
*Experience in simulation*	
None	0 (0)
Little	10 (59)
Moderate	6 (35)
A lot	1 (6)
Experience in anaphylaxis management	
*Number of anaphylaxis cases per year*	
None	1 (6)
1 to 10	11 (65)
11 to 20	3 (17)
More than 20	2 (12)

### Questionnaires

Overall, the simulation experience was positively rated on the 5-point Likert scale, with most participants agreeing that the themes matched their learning objectives and that the level of difficulty was adequate (Fig. 3). Also, 94% (16/17) of the participants considered that the scenarios were representative of what could occur in a clinical setting and eight (8/17, 47%) indicated that they were able to focus during the simulation activity. Eight participants expressed that the simulation activity allowed them to reproduce the same “feelings” they had while managing a severe anaphylactic reaction. Most participants thought that the activity allowed them to identify their strengths (82%) and weaknesses (88%) and to improve their teamwork skills (94%). However, despite steps taken to create a safe learning environment lacking judgment, four participants found the simulation training stressful, and one person indicated feeling somewhat uncomfortable discussing their performance in the group debriefing session. All participants agreed that this activity should be mandatory for all allergy clinic personnel. Three participants spontaneously suggested that this activity should be done once a year to maintain competency.

As can be observed in Fig. 2, after completing the simulation-lab activity, participants showed a significant improvement in their confidence in managing all aspects of acute anaphylaxis except for “code blue” management. The absence of progress for the latter was partly explained by the fact that most of the allergy-immunology fellows, who had recently completed their internal medicine training, considered that their knowledge of code running was appropriate before the activity. One participant indicated lower confidence in performing high-risk challenges and leading a code following the high-fidelity simulation. This same participant suggested that the training met his objectives and should be mandatory.

Finally, in the open questions section, 76% of the participants wrote comments and suggestions indicating that the activity's main strengths were its realism, comprehensive objectives, immediate personalized debriefing, and team-building advantages. Thus, most of the written comments about the experience were positive such as “helped increase my knowledge”, “good scenarios”, and “realistic environment”. The main improvements suggested were that it should be repeated (“minimally once a year”) and that it should last longer and have additional scenarios.

### In situ simulation and impact on code blue management at the allergy clinic

The *in-situ* simulation revealed gaps in the process, especially regarding rapid access to medication and material, code signalling, code team response and crash cart transport. During this practice, a nurse mentioned missing some acute management drugs. A major problem identified was that the code signalling for the outpatient clinic needed to be relayed by multiple intermediates and dispatched to various buildings. During the simulation, the code signalling was never heard in the building where the allergy clinic is located, leading to a significant delay in the crash cart’s arrival. This problem was uncovered during the simulation and resolved with the help of the security team.

In a real code blue management four months after the simulation, the gaps mentioned had been resolved. Notably, access to the medication and the material had been facilitated by adding clear written indications on the walls on where to find different drugs and equipment. A log designed for allergy medication and equipment was added to the acute care room to optimize availability, space and access. New posters indicating how to prepare some rarely used drugs were also added. During the simulation, the recorded time to receive the cart transport was 12 min. During the actual code at our clinic, this delay was reduced to 1 min, representing a 92% improvement.

### Interview

#### Simulation activity

Participants generally agreed that the simulation was a good training activity that allowed them to improve anaphylaxis management in real life and helped them feel more comfortable with diagnosis and interventions in various situations. They agreed that the simulation environment was similar to what they knew from the adult allergy clinic, even if most allergy-immunology fellows (4/5) had not had the chance to manage anaphylaxis before the simulation. They appreciated the practice environment and the quality of the material available, as well as being able to have a hands-on approach. Table 2 summarizes the participant’s positive feedback, identified gaps, and narrative comments.

**Table 2:** Summary of qualitative interviews

Crisis resource management	Positive feedback	Gaps identified	Narrative comments
Communication	Understand the role of other health professionals	Team disorganized during the simulation activity	“*I think it was useful to play the role of the nurse in order to realize the time needed to prepare the different things*”—First-year allergy-immunology fellow
	Improved anaphylaxis management		*“It allowed us to better know each other, to see how others react in stressful situations, and to be able to make mistakes without having a real patient.”*—First-year allergy-immunology fellow
			“*The simulation was very, very helpful for myself, the staff, and the security staff. […] We worked really well together.”*—Allergy-immunologist
Problem-solving	Practice environment		*“The nurses and doctors were efficient and coordinated. The code team collaborated with the allergy team in synergy. We need to congratulate them for all the work. This shows the importance of optimal training.”*—External observer of real-life code management
	Quality of the material available		
	Hands-on approach		
			“*The simulation was very, very helpful for myself, the staff, and the security staff. The simulation was responsible for many improvements, including the rapid intervention of the nurses, and the fact that security was there with the cart in less than a minute and the code team in less than two. We worked really well together.”*—Allergy-immunologist
Resource use	Importance of structured simulation training	Use of sub-optimal simulation equipment	“*It had been more than 10 years since my last case of refractory anaphylaxis in the clinic. I was really happy that we had performed a revision of the procedure and medication before*”—Allergy-immunologist
		Specificities of handling the mannequin	
			“*The material was already opened and had been used before and some parts were non-accessible or missing and, for example, installing an IV line was impossible. (…) We are used to regularly taking vital signs in an acute situation and the material used during the simulation was different, including the monitor that showed the vital signs, which made the situation a bit confusing*.”—Allergy nurse
			“*Conjunctivitis, rhinitis, skin eruption and signs are difficult to reproduce on a dummy but are important elements to get the feeling of where the reaction is heading in real life”.*—Second-year allergy-immunology fellow

Some nurses found it more difficult to naturally fill their roles because of the equipment available and the specificities of handling the mannequin. This comment was echoed by one of the allergy-immunology fellows, who mentioned the limitations of the mannequin in simulating clinical signs of anaphylaxis. Despite these limitations, the activity was appreciated, and there was a consensus that the activity increased confidence and reassurance in the allergy clinic.

The participants underlined essential elements of team-building. It was felt that the simulation helped the team to “learn to work together.” It clarified expectations and therefore helped team members to trust one another. This was made possible by the safe environment provided by the simulation, where participants felt comfortable making mistakes.

#### Code blue management

Overall, the interviews revealed that simulation-based training led to more rapid and orderly responses and improved confidence in the participants’ abilities and colleagues’ abilities in managing anaphylaxis. During the actual code blue management, the team was able to stabilize the patient and improvement was noted in various aspects of the process compared to the *in-situ* simulation. The physician and nurses involved in the code felt an improvement in the team dynamic and physical environment following the simulation-based training. The managing physician added that he considered that the training should be done annually for the physicians and the staff because of the paucity of severe refractory reactions.

#### Management of other real-life anaphylaxes in the clinic

Regarding anaphylaxis management, a nurse found that the team had sometimes been “disorganized” during the simulation. Still, during a subsequent reaction in the clinic, this same participant indicated that “everything was methodical, and everyone’s role was clear.” One of the physicians echoed this, who mentioned that the staff was “very calm” when managing mild to moderate anaphylaxis.

Three allergy-immunology fellows also mentioned feeling more structured and confident in recognizing anaphylaxis and administering epinephrine. They also agreed that, in general, the staff was “efficient,” “the medication was easily accessible,” and that the health professionals and patients were “more confident and reassured” when confronted with an anaphylaxis reaction.

### Discussion

#### Key findings

Critical care simulation in anaphylaxis at our center allowed participants to identify their strengths and weaknesses and improve their teamwork skills. By conducting post-activity questionnaires and interviews, participants indicated an improvement in several aspects of crisis and anaphylaxis management. The simulation identified a critical gap regarding code blue signalling in the new building and other gaps in the process, such as access to drugs and materials, which were later improved. Overall the activity was very much appreciated, and the participants considered that it should be a mandatory yearly training opportunity.

### Previous studies

Similar reports targeting medical and administrative personnel from the community and hospital-based allergy clinics have assessed teaching and retention of emergency management team skills using high-fidelity mannequins, standardized patients and, 10–12 months after the activity, an unexpected in situ simulation [1, 15]. These studies showed improved team management skills in areas such as teamwork and situation awareness, as well as retention of knowledge and abilities after an initial anaphylaxis scenario workshop [15]. Similar studies focused on implementing and using an anaphylaxis and allergy-immunology emergencies simulation curriculum for allergy-immunology trainees [15, 16].

The literature on multidisciplinary team dynamics in anaphylaxis is scarce. In one of the studies mentioned above, the authors focused on the importance of engaging the medical and non-medical personnel to clarify their specific roles to avoid confusion and repetition [1]. In our study, the non-medical personnel were also present during the *in-situ* simulation allowing them to witness a severe anaphylaxis management scenario firsthand. In the more general acute settings such as the emergency department, the operating room and the intensive care unit, there has also been an interest in characterizing team-based simulation [5]. A review paper including 17 studies underlined the importance of this team training program model aimed at increasing authenticity and improving patient care at an administrative level [5].

Similarly, a systematic review of 38 articles on simulation activities, including 22 randomized controlled trials, found that individual and team performances were improved during critical events and complex procedures [17]. Our results showed a perceived improvement in crisis team management, and 94% of the participants considered that this activity allowed them to improve their teamwork skills. These essential team-building elements were also reported during the interviews. Similarly, medical education programs should focus on developing simulation training to ensure teamwork skill-building through practice and repetition [3, 18].

The questions concerning participants’ confidence before the activity revealed that some staff members had insecurities regarding the appropriate management of anaphylactic reactions. We showed that confidence could improve after simulation training. This was also reflected in the interviews, where participants reported an improvement in their own and other staff members’ ability to manage anaphylaxis. Some studies focusing on emergency responses shared similar conclusions with statistically significant improvement in participants’ confidence after a simulation scenario [19, 20]. One participant reported decreased confidence in code management or performing high-risk challenges. In light of other answers given by the same participant, this seems to be explained by the discovery of unsuspected knowledge gaps, which led the participant realizing that they were not as performant as they would have liked.

The *in-situ* simulation proved essential for identifying and solving gaps in the process that could not be captured during lab simulation (access to material and medications, code signalling, and intensive care response). The main benefits of an actual medical setting simulation described in the literature are the possibility to evaluate participants’ knowledge and competencies and the clinical environment to improve patient safety [4, 5, 7].

### Limitations

This study has limitations. It was performed in a single institution with a limited number of participants. Implementing this type of simulation in other allergy clinics requires considering numerous factors, such as clinic space, material distribution, and staff experience, which are expected to vary between centers. Access to a high-fidelity simulation lab and costs are essential barriers that could prevent the reproducibility of the activity. Here, the recent clinic relocation was used to justify the need for the activity. While all agree that patient safety is paramount, it must be clarified to what extent improved team functioning, efficiency and quality of care resulting from the activity can offset the costs of a simulation-based training. Another significant limitation is that the conclusions of this article are based on a qualitative assessment of the participant’s perceptions.

Furthermore, the perception of confidence should have ideally been measured before and after the intervention. In our study, this was measured following the intervention, which could bias participants’ responses. It did not objectively demonstrate improvements in patient outcomes attributable to the activity, which would have required a prospective experimental design looking at patient outcomes or crew resource management skills assessed by an external observer [21]. Quantifying the value of this qualitative benefit represents an important area of future research [17].

### Implications

In an era where virtual reality is increasingly used as simulation technology, it is essential to describe our simulation program’s success and underline its benefits for inter-professional collaboration and patient care.

### Conclusion

This study provides critical qualitative data supporting the positive impact of a high-fidelity anaphylaxis training activity on anaphylaxis management in the clinical practice. Participants deemed the activity instrumental in improving staff readiness and decreasing reluctance to perform challenges or procedures at high risk of anaphylaxis in the ambulatory setting. It provides further evidence that high-fidelity simulations should be included in the continuous medical education curriculum for allergy-immunology specialists to improve patient safety and team confidence. Other studies are required to guide best teaching practices using tools such as high-fidelity simulation to manage acute allergic reactions.

## Data Availability

The datasets used and analyzed during the current study are available from the corresponding author upon reasonable request.

## Abbreviations

ACLS: Advanced cardiac life support
BLS: Basic life support
CHUM: Centre Universitaire de l’Université de Montréal
CanMEDS: Canadian Medical Education Directions for Specialists
